# Study of the Durability of Membrane Electrode Assemblies in Various Accelerated Stress Tests for Proton-Exchange Membrane Water Electrolysis

**DOI:** 10.3390/ma17061331

**Published:** 2024-03-14

**Authors:** Zhengquan Su, Jun Liu, Pengfei Li, Changhao Liang

**Affiliations:** 1 Institute of Physical Science and Information Technology, Anhui University, Hefei 230601, China; q21201318@stu.ahu.edu.cn; 2 Key Laboratory of Materials Physics, Anhui Key Laboratory of Nanomaterials and Nanotechnology, Institute of Solid State Physics, Chinese Academy of Sciences, Hefei 230031, China; pfli@issp.ac.cn (P.L.); chliang@issp.ac.cn (C.L.)

**Keywords:** accelerated stress tests, PEM water electrolysis, step time, square-wave, voltage rise, membrane electrode assemblies

## Abstract

In this work, we focus on the degradation of membrane electrode assemblies (MEAs) in proton-exchange membrane water electrolysis (PEMWE) induced by different accelerated stress tests (ASTs), including constant-current mode, square-wave mode, and solar photovoltaic mode. In constant-current mode, at continuous testing for 600 h at 80 °C, a degradation of operating voltage increased by the enhanced current density from 22 µV/h (1 A/cm^2^) to 50 µV/h (3 A/cm^2^). In square-wave mode, we found that in the narrow fluctuation range (1–2 A/cm^2^), the shorter step time (2 s) generates a higher degradation rate of operating voltage, but in the wide fluctuation range (1–3 A/cm^2^), the longer step time (22 s) induces a faster operating voltage rise. In the solar photovoltaic mode, we used a simulation of 11 h sunshine duration containing multiple constant-current and square-wave modes, which is closest to the actual application environment. Over 1400 h ASTs, the solar photovoltaic mode lead to the most serious voltage rise of 87.7 µV/h. These results are beneficial to understanding the durability of the PEM electrolyzer and optimizing the components of MEAs, such as catalysts, membranes, and gas diffusion layers.

## 1. Introduction

The extensive usage of fossil fuels has exacerbated environmental issues, intensifying the urgent need to develop alternative energy sources. In line with the swift progress in wind and solar photovoltaic power installation, the development of suitable energy storage solutions is imperative [1]. In a perfect scenario, the combination of water decomposition and renewable energy sources could offer a practical approach to converting excess energy into hydrogen, a promising solution for both renewable energy storage and fluctuation challenges [2,3,4]. Proton-exchange membrane water electrolysis (PEMWE) stands out as a leading method for clean hydrogen production due to its compact design, high efficiency, and operational flexibility, which enable its direct coupling with renewable energy sources [5]. Nevertheless, the intermittency of and fluctuation in renewable energy sources can degrade the performance of proton-exchange membrane water electrolysers, potentially leading to increased voltage values in the electrolyzer [6]. Aiming at this issue, the challenge lies in effectively assessing durability under laboratory conditions and identifying the critical degradation parameters, particularly during dynamic operation, which is not yet well understood [7,8].

The operational lifespan of PEMWE have been the subject of numerous studies in the literature [9,10,11,12,13,14]. It has been well accepted that both constant-current (static) mode and square-wave (dynamic) mode may lead to performance degradation. The overall performance degradation of an electrolyzer mainly comes from the degradation of the MEA, which results from two sources: the proton-exchange membrane and the catalyst. A proton-exchange membrane could degrade or become contaminated, which would result in degradation of the electrolytic performance. Membrane degradation is divided into three categories: mechanical, chemical, and thermal degradation. Of these, chemical and thermal degradation are more affected by fluctuations in the power supply. In the anode of an electrolyzer, water undergoes an oxidation reaction, and H_2_O_2_ or hydroxyl radicals generated through the two-electron reaction pathway attack the hydrocarbon chain segments of the perfluorinated sulfonic acid membrane, causing the membrane to degrade [10]. When faced with fluctuating conditions, the uneven distribution of current in the catalyst layer produces a local hot area that causes uneven heating of the proton-exchange membrane, resulting in membrane expansion or deformation, and at the same time, the small amount of hydrogen and oxygen that passes through the membrane reacts with exothermic heat [15], which also produces a local hot area, thus accelerating the degradation reaction of the membrane. And catalyst degradation can be further categorized into redeposition, Ostwald ripening, and coalescence degradation [16,17]. In the constant-current mode, when operating at a current density of 3 A/cm^2^ for 1000 h, it was observed that the corrosion rate at 3 A/cm^2^ was triple that at 1 A/cm^2^ [18]. Chandesris et al. [19] have discovered that variations in current density had a negligible impact on attenuation under constant-current test conditions of 10 A/cm^2^. However, this constant-current operation does not accurately reflect the actual conditions of PEMWE application. Li et al. [20,21] have conducted experiments with low-current fluctuations ranging from 0 to 0.5 A/cm^2^ and 1.2 to 2 A/cm^2^, demonstrating that low-current cycling had a more significant effect on voltage degradation, and that a reduction in ohmic losses under high-current cycling indicated membrane thinning and failure. Rakousky [22,23] and Fouda-Onana [24] have attributed the degradation to the oxidation of the porous titanium plate, which serves as a transport layer, as well as the thinning of the polymeric membrane. Rakousky et al. [25] have investigated PEMWE with five different current density fluctuation curves and identified varying degrees of degradation. Steffen et al. [26] have systematically examined the impact of different dynamic modes on the degradation of PEMWE, finding that faster current cycling enhanced the overall performance over a test duration of 500 h. Despite this, the precise degradation mechanisms [27,28,29,30,31,32] that impact their performance remain largely unexplained. Furthermore, there is ongoing ambiguity regarding the differential influence of various current modes, such as the dynamic rate of the current on the degradation rates.

As the MEA production process continues to advance, performance and stability have been improved, and in order to assess the stability of the new MEA, it is necessary to run it for a longer period of time, which undoubtedly greatly increases the time cost. For MEAs with excellent stability performance, it is more difficult to observe the performance in the later period of the test, so some extreme test conditions are needed to accelerate the aging of MEA, so that the MEA can reach the state in the later period of the test in a relatively shorter period of time. Aiming at this issue, we have designed three acceleration schemes in the hope of finding the factor that causes the rapid degradation of MEA performance in electrolyzer. The three acceleration schemes are constant-current mode for 600 h, square-wave mode for 600 h, and simulating solar PV mode over 1400 h, which are used in this work to investigate the effects of current density, fluctuation range, and step time on the voltage rise of a PEM electrolyzer. Corresponding EIS investigations were performed to explain the very reason for the degradation rate of MEAs.

## 2. Experimental

### 2.1. Membrane Electrode Assemblies and Test Bench Setup

The membrane electrode assemblies (MEAs) were provided by Anhui Contango New Energy Technology Co., Ltd. (Hefei, China), where Pt/C with a loading of 0.5 mg/cm^2^ and Ir black with a loading of 1.0 mg/cm^2^ were used as cathode catalysts and anode catalysts, respectively. The PEM membrane with a thickness of 120 µm (DM6321A) was from Dongyue Future Hydrogen Energy Co., Ltd. (Zibo, China) Carbon paper (TGP-H-60, Toray, Tokyo, Japan) with a thickness of 0.18 mm was employed as the cathode gas diffusion layer (GDL), and Pt-coated porous sintered titanium was used as the anode GDL. The cathode end-plate had serpentine-shaped flow fields and the anode end-plate had dotted flow fields. The effective active area of the MEA in all the experiments was 30.624 cm^2^ (5.80 cm × 5.28 cm). The single-cell electrolyzer was secured with screws and nuts, and the amount of compression was set at 2.7 MPa for this experiment. The structure of the electrolyser used in this experiment is shown schematically in Figure S1. The single-cell electrolyzer was connected to a 6 mm inner diameter water flow line, and a pump continuously supplied deionized water to the PEMWE at a flow rate of 100 mL/min to circulate the deionized water between the tank and the PEMWE anode and replenish the tank with deionized water at regular intervals in order to minimize the adverse effects of the decrease in water on the membrane electrodes as a result of the increase in the concentration of ions. A variety of current switching processes were implemented on the PEMWE system with a temperature of 80 °C for durability test.

### 2.2. Electrochemical Measurements

**Figure 1:**
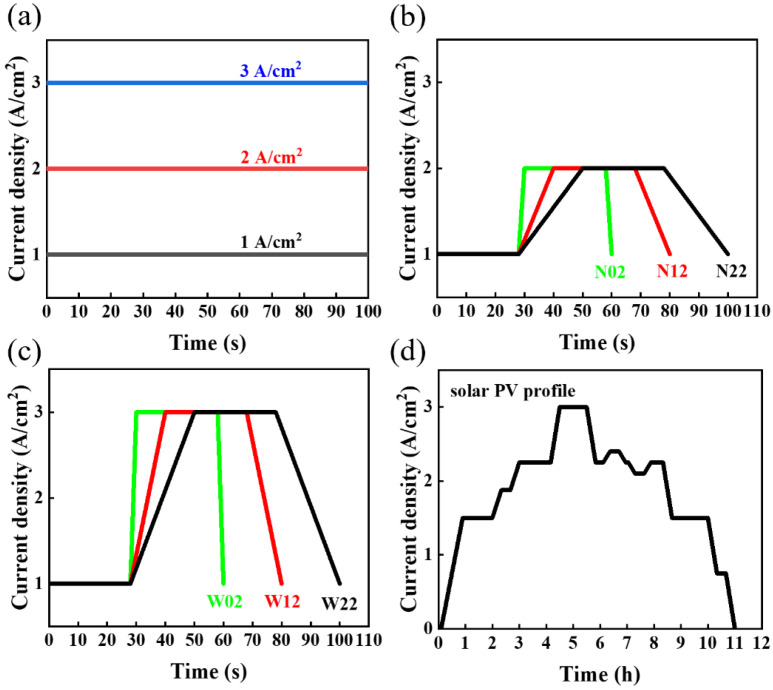
The AST waveforms used in this investigation. (**a**) The 1, 2, and 3 A/cm^2^ constant-current density mode tests. (**b**) Narrow square-wave mode test with fluctuations in the range of 1–2 A/cm^2^. (**c**) Wide square-wave mode test with fluctuations in the range of 1–3 A/cm^2^. (**d**) Simulating solar PV fluctuation mode within a current density range of 0–3 A/cm^2^.

Electrochemical impedance spectroscopy (EIS) measurements were performed in yhr constant-current mode at a frequency range between 10 kHz and 1 Hz. The cyclic voltammetry curve was measured at a scan rate of 0.01 V/s over a voltage range of 0.05–1.3 V. More detailed experimental procedures of this investigation are displayed in Figure S2. The durability experimental tests were performed by a computer-controlled DC power supply, temperature probe, voltmeter, impedance meter, and electrochemical workstation, which contained the following steps:

(1) Activation process: Before the performance test, an activation procedure was applied to the electrolyzer using ITECH’s DC power supply IT-M3110 (ITECH, Nanjing, China) to provide current, and it was carried out in the following order: 0.2 A/cm^2^ for 1 h, 1 A/cm^2^ for 1 h, 2 V for 0.5 h, 1.7 V for 2 h, and 2 V for 0.5 h.
(2) Accelerated stress tests (ASTs): Three types of ASTs were performed. The first one was the constant-current mode test at 1 A/cm^2^, 2 A/cm^2^, and 3 A/cm^2^; The second was square-wave mode tests fluctuating in the range of 1–2 A/cm^2^ and 1–3 A/cm^2^; a cycle of the square-wave mode consisted of two parts, a current-holding part and a current-change part. In this investigation, the current-holding part was used for 28 s dwell time, while the current-varying part was used for different step times (2 s, 12 s, and 22 s). The last AST simulated a solar fluctuation, using the power generated in Qinghai, China, during 13 h of sunshine on a day in June 2022. Detailed parameters of the ASTs are displayed in Table 1, and the corresponding test waveforms are shown in Figure 1a–d.

## 3. Results and Discussion

This section compares the degradation in different operation modes; however, in order to better understand the dynamic mode of operation of the grid service and discuss the impact of transient changes in PV power generation on the electrolyzer, the analysis focuses on the discussion of the AST, which has faster degradation rates: N02 and W22. Although, none of them decay as fast as Solar, and the degradation rates for each AST are shown in Table 2.

### 3.1. PEMWE Performance Change

This investigation is divided into three modes: constant current, square wave, and simulated solar. In the 600 h test time of this experiment, except for the existence of performance enhancement at the beginning of the test, the voltage gradually increases with time thereafter.

**Figure 2:**
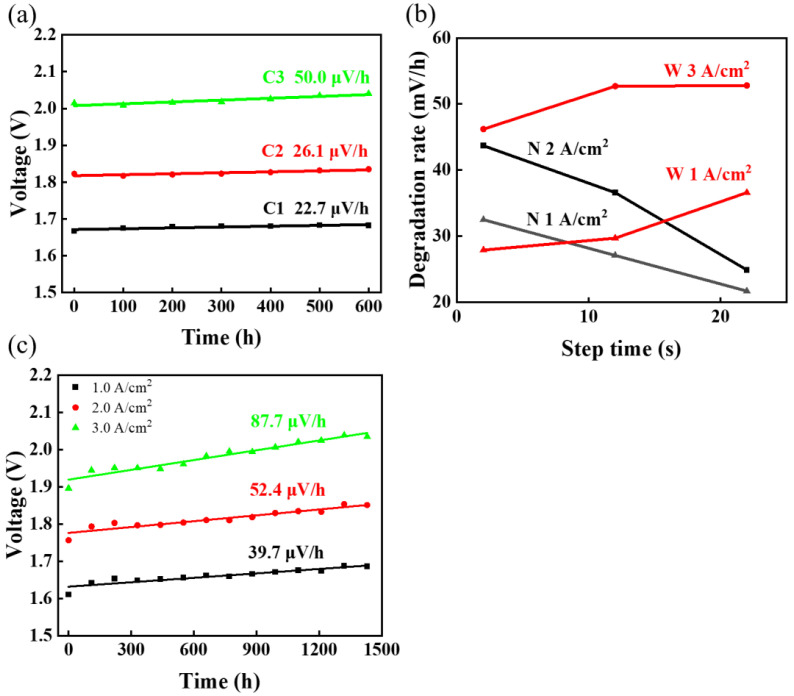
(**a**) Voltage change at different constant-current density, and the slope of the fitted line represents average degradation rate. (**b**) The relationship between degradation rate and step time in narrow and wide square-wave tests. (**c**) Voltage rise in solar at different current densities.

Figure 2 shows that the degradation rates of different modes are obviously different. Figure 2a shows the degradation pattern in the constant-current mode, and the three fitted lines show the degradation rates of the three constant currents. Consistent with common sense, a higher current leads to a greater degradation rate. Figure 2b shows the relationship between the degradation rate and the step time for different square-wave fluctuations, and the relationship between the degradation rate and the step time appears to be different for the wide and narrow square-wave tests. The plots of the voltage with time in different square-wave tests are compared, as shown in Figure S3. Briefly, the degradation rate is linearly correlated with the step time, although this trend changes depending on whether a narrow or wide square-wave test is used. Increasing the step time in the wide square-wave test would accelerate degradation; conversely, increasing the step time in the narrow square-wave test would slow degradation. Figure 2c demonstrates that the largest increase in voltage in this investigation is solar. And the data for all sets of voltage over time are shown in Tables S1–S4.

**Figure 3:**
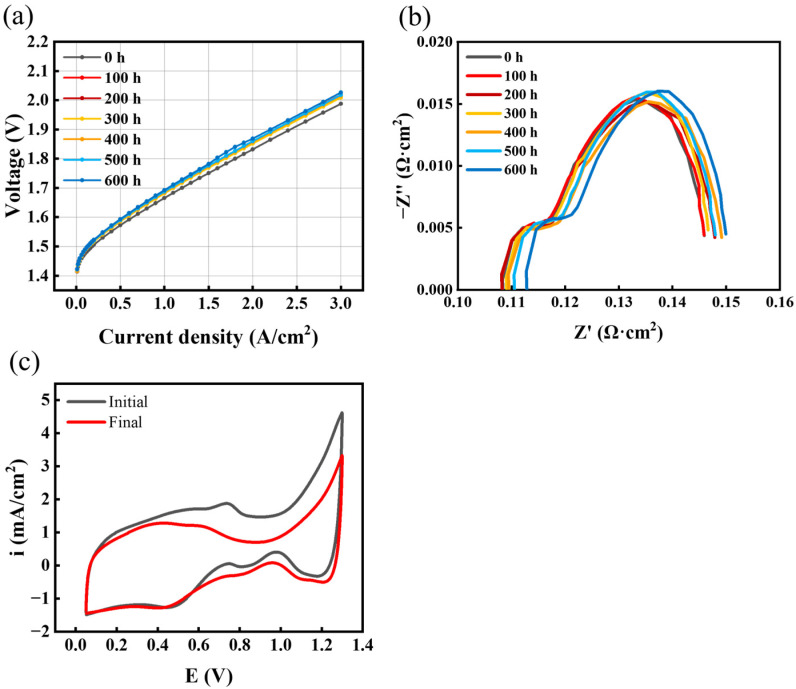
C3′s (**a**) polarization curves at different times, (**b**) EIS over time, and (**c**) CV plots changes before and after AST.

More information could be derived from Figure 3a–c, as after AST, the degradation rate at the larger current was always greater than the degradation at the smaller current. The degradation rate follows a different pattern from the current step time for different square-wave modes. In the wide square-wave mode, the degradation rate of W02 increased from 46.2 μV/h to 52.7 μV/h with W12, continued to increase the current step time W22, and then degradation rate reached a maximum value of 52.8 μV/h calculated at 3 A/cm^2^. The rate of degradation increased with the increase in the current step time. In contrast, the degradation rate decreased with the increasing step time in the narrow square-mode test. The speculation of this phenomenon is that in the narrow square-wave mode, low current would produce less impact, and the main influencing factor of degradation would be the rapid change in current; meanwhile, in the wide square-wave mode, it would be exposed to higher currents for longer time, the main influencing factor would be the high current, and the degree of impact of the high current would be greater than the current switching process, which explains the increase in the current step time accompanied by an increase in the decay rate. Therefore, this leads the fastest AST obtained from the square-wave mode to be W22.

The constant-current mode test was used to allow the electrolyzer to run at 1 A/cm^2^, 2 A/cm^2^, and 3 A/cm^2^ for 600 h. Polarization curves and EIS were recorded periodically to analyze the degradation pattern. Figure 2a indicates that the fastest AST in the constant-current mode is C3, and Figure 3 shows the electrochemical tests during the test period. Figures S4 and S5 show the difference between the fresh MEA and the MEA after running at a constant-current density of 3 A/cm^2^ for 600 h, where more cracks and bumps were observed on the surface of the anode catalyst layer by using SEM. Additionally, the content after EDS showed a change in the elemental content, mainly a decrease in the percentage of Ir from 86.62% to 81.71%, an increase in O from 7.79% to 10.45%, and in increase in F from 5.59% to 7.84%. A similar situation occurred with the square-wave mode test and the solar photovoltaic mode ASTs, as shown in Figures S6–S8 (N02, W22, and solar). The CV curves in Figure S9, observed from the initial (0 h), intermediate state (200 and 400 h), and final (600 h) states, show a larger recession in AST at W22, which is consistent with the larger increase in measured voltage.

The IV curve in Figure 3a shows that in the part of the current density that is less than 0.5 A/cm^2^, the polarization curve basically does not change with time, indicating that the electrolyzer performance is stable and less affected by test condition. However, in the region of higher current density, the voltage gradually increases with time. Although during the early part of this investigation the magnitude of voltage values increased and decreased at times, all the values showed a pattern of degradation after 600 h of AST. Figure 3b shows the Nyquist plot of the EIS spectrum at 0.2 A/cm^2^; the high-frequency impedance decreases in the early part of AST and then increases, which is consistent with the voltage decrease in the polarization curve in the early part of the AST. The initial and final CV curves of the fastest AST for the constant-current mode are shown in Figure 3c. Usually, the area under the CV curve represents the amount of redox charge involved in the electrochemical reaction. The capacitance current will be less than the initial current, and this is a result of the lower performance of the electrolyzer; the same change has been observed in previous studies [33,34]. The average oxidation and reduction currents of the electrolyzer decreased after the durability test, the catalytic performance decreased, and the degradation was evident in the electrochemical characterization tests.

**Figure 4:**
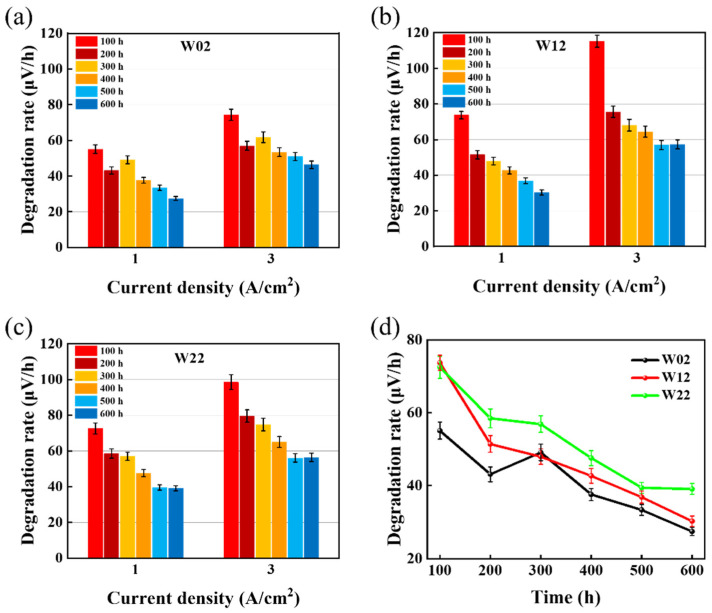
Average degradation rate at 1 A/cm^2^ and 3 A/cm^2^ for (**a**) W02, (**b**) W12, and (**c**) W22 with time in wide square mode. (**d**) Comparison of the degradation of three ASTs at 1 A/cm^2^ in wide square-wave mode.

Figure 4 shows the degradation of the three ASTs in wide square-wave mode, with the average degradation rate calculated every 100 h for a total of 600 h of degradation. The average degradation rate at different times of the wide square mode can be seen from Figure 4a–c, except for the 100 h at the beginning of the experiment. Due to other factors, W12 has a faster degradation in the same fluctuation range, and then all of the wide square-wave modes satisfy the pattern that the degradation rate and the current step time are positively correlated. Passivation of Ti tends to occur at high potentials because when the titanium capacitive is passivated and an oxide layer is formed on the surface, the oxide layer prevents the transfer of electrons and ions, leading to an increase in electrolytic resistance, which in turn requires a higher voltage to maintain a certain current density [35,36]. This will increase the energy consumption and voltage of the electrolysis process. After the titanium plate passivates to a certain degree, the surface oxide layer will not be significantly thickened by the high voltage; that is, the titanium plate reaches a more stable state due to the existence of the surface oxide layer, which enhances the corrosion resistance and electrochemical stability of the titanium plate at the expense of part of the electrode efficiency to obtain the property of long-term stability. However, since this part of the change occurs inside the electrolyzer, there is no study that can accurately describe the mechanism of the change. Some relevant studies have shown that it is possible to plate a layer of titanium plate with a good conductivity line and electrocatalytic activity Pt, the purpose of which is to artificially inhibit the oxidation of the titanium plate and protect the Ti substrate from corrosion passivation with the Pt layer, which can reduce the electrolysis voltage to a certain extent and improve the performance of the electrolytic water by enhancing the stability of the components.

### 3.2. Analyzing the Evolution of Resistance

**Figure 5:**
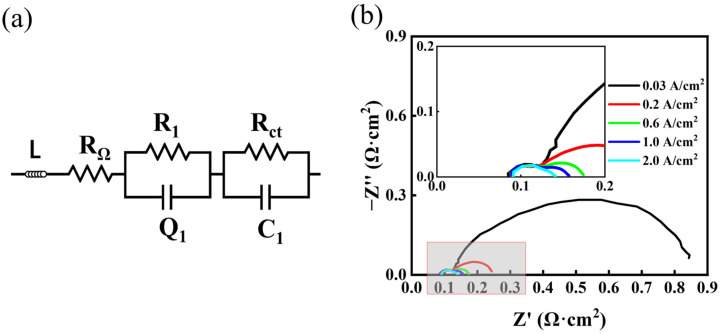
(**a**) Equivalent circuit model [40] and (**b**) EIS measured at different current densities.

As can be seen in Figure 5b, an impedance spectrum can be described by two semicircles, which correspond to the two R-C models in Figure 5a and are referred to as the high-frequency arc R_HF_ and the low-frequency arc R_LF_, whose centers are below the real axis. This indicates the presence of non-ideal capacitive behavior [37,38]. The high-frequency intercept on the real axis represents the total ohmic resistance of the electrolyzer, mainly from the electrical or ionic conductive elements, which corresponds to R_ohm_ in the equivalent circuit model. The difference between the low-frequency and high-frequency intercepts of Rohm in the plot is defined as the polarization resistance (R_p_). The shape and magnitude of the impedance spectrum of the electrolytic bath significantly change with current density. When the current density is high enough, the polarization resistance (R_HF_ + R_LF_) becomes small and the impedance becomes mainly ohmic. The literature suggests that the effect of the cathode process is responsible for R_HF_ [39]. On the other hand, the anode process (OER) corresponds to R_LF_. Since the analysis of impedance at low currents is of practical interest because of the possibility of partial-load operation in grid-balancing services and the phenomena observed at small current densities mainly reflect the behavior of the catalyst, comparatively, the AC impedance at high current densities is not very useful.

The loss of performance is categorized into reversible loss and irreversible loss, and it has been shown that allowing the electrolyzer to dwell time (zero current) for a sufficient period of time could restore some of the reversible loss [41]. In addition to reversible loss, destructive degradation of the catalyst occurs, which results in an increase in the polarization resistance, known as irreversible loss. Apart from the effect of the catalyst, other factors that contribute to the voltage increase include the decline in individual components in the electrolyzer.

The relevant literature indicates that the changes in electrolyzer performance can be explained by the changes in the proton exchange membrane and PTL, and the effect of PTL is mainly a negative effect due to surface corrosion and the passivation of the Ti plate, but there may also be other effects on non-ohmic resistance. The common method used today is the Nyquist plot, where the high-frequency impedance represents the total ohmic impedance. In this investigation, the high-frequency impedance was analyzed to understand the change in ohmic impedance of the membrane electrodes in different ASTs.

**Figure 6:**
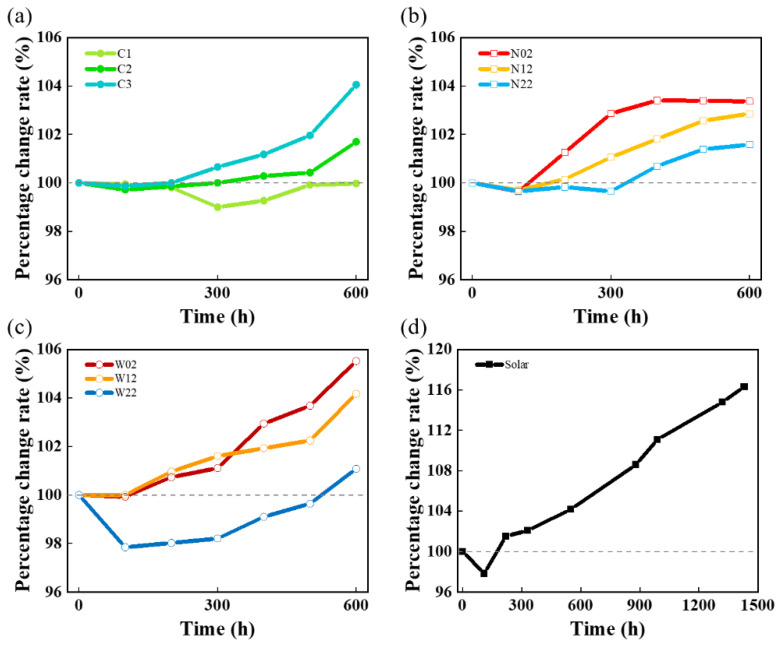
Percentage change rate in HFR over time for (**a**) constant-current mode, (**b**) narrow square-wave mode, (**c**) wide square-wave mode, and (**d**) solar.

**Figure 7:**
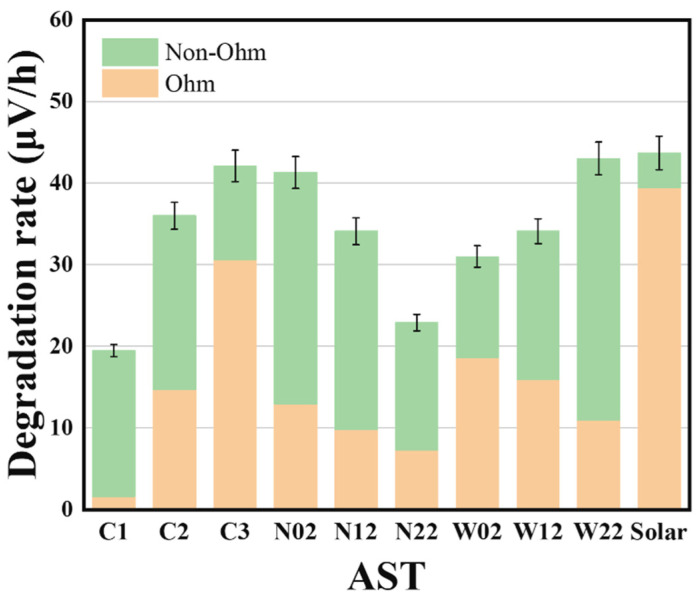
Separation of the impact of ohmic resistance on the voltage degradation rate at 2.0 A/cm^2^. The total degradation rate was extracted from IV curves at different times.

Figure 6 shows the percentage rate of change in high-frequency impedance with time, at a current density of 2 A/cm^2^. In this investigation design of ASTs, a slight decrease in high-frequency impedance at the beginning of the test can be observed. We speculate that it is the insufficient activation process that produces this phenomenon, so all the first 100 h are regarded as the activation time in the impedance calculation process; thereafter, the increase in high-frequency impedance was mainly due to the effect of the titanium plate [18,25]. By analyzing the graphs as the ohmic resistance increases, the degradation rate due to a combination of influences other than the titanium plate can be obtained. The total degradation rate was extracted from the polarization curve, except for first 100 h at 2 A/cm^2+^, and HFR increasing was used to calculate the degradation from the titanium plate, which was subtracted to obtain the non-ohmic degradation. From Figure 7, although the pattern of total degradation rate versus current step time varied with square-wave mode, evidently, as the step time increases, the degradation rate due to ohmic impedance, i.e., the negative impact of corrosion and passivation on the performance of the titanium plate, decreases. This was probably due to the shorter residual time of oxygen within the electrode operation during cyclic operation. This is also the reason suggested earlier as a reason for lower local membrane dehydration of the proton membrane dehydration.

Compared with the previous ASTs in this investigation, the ohmic impedance follows the same law of becoming smaller and then increasing. Because the durability test time is long for 1430 h, and the ohmic impedance decrease was observed in the first 100 h, which indicates that the passivation of the Ti plate was basically stable, the decrease in the ohmic value in the early part of the investigation was caused by the incomplete activation process.

The degradation rates shown in Figure 6a are consistent with common sense, and high current will promote degradation. C1 is special, as the final high-frequency impedance has a very small reduction compared to the start, indicating that the influence of C1 on the titanium plate is small. Alongside this, the membrane and the titanium plate bring about the effect to counteract each other, so the 1 A/cm^2^ current mode is not able to accelerate degradation. Figure 6b,c shows the percentage change in high-frequency impedance in the square-wave mode, and larger changes occur in the wide square-wave mode, affected by high current. Meanwhile, Figure 6d illustrates that solar simulation would cause more severe corrosion and passivation of the titanium plates, in the form of increased high-frequency impedance.

Figure 7 shows that the separation of voltage degradation rate and the phenomenon of high-frequency impedance decrease exist in the first 100 h of each AST, indicating that the electrolyzer has not reached a stable state; consequently, it is not included in the calculation of the degradation rate separation. Thereafter, the high-frequency impedance continues to increase, implying that the titanium plate is gradually corroding and passivating. Interestingly, different from the law of total degradation rate, the voltage degradation due to ohmic resistance decreases when increasing the step time. The speculation of this phenomenon is that the gas produced from the catalyst layer is not discharged in time under the case of rapid current change and remains in the GDL; thus, the insufficient exchange of substances increases the impedance value, which ultimately leads to irreversible damage to the membrane electrodes.

## 4. Conclusions

In summary, we investigated the impact of different AST conditions on an electrolyzer’s performance, including constant-current, square-wave, and simulate photovoltaic fluctuations, by collecting polarization curves and electrochemical impedance spectroscopy data, offering comprehensive insights into the electrolyzer’s performance dynamics.

In the constant-current density mode tests, a consistent consensus was that the higher current density yields higher degradation rates of the operating voltage compared to the instances involving lower current densities. The square-wave measurements lead to two counter trends: within a narrow fluctuation range (1–2 A/cm^2^), the short step time (2 s) exhibits a higher degradation rate compared to the long step times (12 and 22 s). However, within a wide fluctuation range (1–3 A/cm^2^), the trend is inverse. The degradation rate associated with the current step time of 22 s was observed to be higher than those of the 2 and 12 s. For the simulating solar test, which consists of multiple constant-current density and square-wave modes, the investigations indicate that a high current density and long step time would lead to the most serious degradation rate compared with other ASTs.

Our investigation offers valuable insights into accelerating test efficiency and understanding service life by elucidating the intricate relationship between current-related parameters and electrolyzer performance degradation. Understanding these factors makes it possible to balance research with the design and operation of PEM electrolysis systems to improve overall performance and reliability. Consequently, when executing ASTs that encompass high-current conditions, meticulous consideration and balance of all component responses become imperative. This equilibrium is crucial due to the interconnected nature of the system components.

## Figures and Tables

**Table 1:** Investigated operation modes. Each AST was kept running under the same test conditions for 600 h, except the solar PV profile which was running for 1430 h.

Operation Mode	Test Condition	Mode of Change (Name)
Constant current	600 h, 80 °C	1 A/cm^2^ (C1)
		2 A/cm^2^ (C2)
		3 A/cm^2^ (C3)
Narrow square wave	1~2 A/cm^2^ 28 s dwell time 600 h, 80 °C	2 s step time (N02)
		12 s step time (N12)
		22 s step time (N22)
Wide square wave	1~3 A/cm^2^ 28 s dwell time 600 h, 80 °C	2 s step time (W02)
		12 s step time (W12)
		22 s step time (W22)
Solar photovoltaic mode	0~3 A/cm^2^ 1540 h, 80 °C	Simulating fluctuation (Solar)

**Table 2:** Degradation rate analyses for different ASTs used in this investigation.

Test Mode	AST	Degradation Rate	Impact on Degradation
Constant-current mode	C1	22. 7 μV/h @ 1 A/cm^2^	High current density would accelerate degradation.
	C2	26.1 μV/h @ 2 A/cm^2^	
	C3	50.0 μV/h @ 3 A/cm^2^	
Narrow square-wave mode (1–2 A/cm^2^)	N02	32.5 μV/h @ 1 A/cm^2^ 47.3 μV/h @ 2 A/cm^2^	Fluctuates in the range of 1–2 A/cm^2^, decreasing step time of current would accelerate degradation.
	N12	27.1 μV/h @ 1 A/cm^2^ 36.6 μV/h @ 2 A/cm^2^	
	N22	21.7 μV/h @ 1 A/cm^2^ 24.9 μV/h @ 2 A/cm^2^	
Wide square-wave mode (1–3 A/cm^2^)	W02	27.9 μV/h @ 1 A/cm^2^ 46.2 μV/h @ 3 A/cm^2^	Fluctuates in the range of 1–3 A/cm^2^, increasing step time of current would accelerate degradation, but there seems to be an upper limit.
	W12	29.8 μV/h @ 1 A/cm^2^ 52.7 μV/h @ 3 A/cm^2^	
	W22	36.7 μV/h @ 1 A/cm^2^ 52.8 μV/h @ 3 A/cm^2^	
Simulating solar mode	Solar	39.7 μV/h @ 1 A/cm^2^ 52.4 μV/h @ 2 A/cm^2^ 87.7 μV/h @ 3 A/cm^2^	Near 2–3 A/cm^2^, increasing step time, the maximum degradation rate occurs.

## Data Availability

Data are contained within the article and Supplementary Materials.

## Supplementary Materials

The following supporting information can be downloaded at: https://www.mdpi.com/article/10.3390/ma17061331/s1. Figure S1. A graphical outline of accelerated stress tests. Figure S2. (a) Schematic diagram of the structure and (b) exploded view of the electrolyzer used in the investigation. Figure S3. Degradation rate of narrow square wave mode in (a) 1 A/cm^2^ and (b) 2 A/cm^2^, and degradation rate in (c) 1 A/cm^2^ and (d) 3 A/cm^2^ for wide square wave mode, the slope k represents the degradation rate. Figure S4. (a) High and (b) low magnification SEM images and (c–f) EDS mappings of the fresh MEA used in this investigation. Figure S5. (a) High and (b) low magnification SEM images and (c–f) EDS mappings of the MEA after constant current mode AST C3. Figure S6. (a) High and (b) low magnification SEM images and (c–f) EDS mappings of the MEA after narrow square wave mode AST N02. Figure S7. (a) High and (b) low magnification SEM images and (c–f) EDS mappings of the MEA after wide square wave mode AST W22. Figure S8. (a) High and (b) low magnification SEM images and (c–f) EDS mappings of the MEA after solar photovoltaic mode AST. Figure S9. CV curves over time in (a) constant current density mode AST C3, (b) narrow square wave mode AST N02 and (c) wide square wave mode AST W22. Table S1. Voltage at different times in constant current test. Table S2. Voltage in 1 A/cm^2^ and 2 A/cm^2^ at different times in narrow square wave mode test. Table S3. Voltage in 1 A/cm^2^ and 3 A/cm^2^ at different times in wide square wave mode test. Table S4. Voltage at different times in Simulating solar fluctuation test.
