# Supplementary material for: Study of the Durability of Membrane Electrode Assemblies in Various Accelerated Stress Tests for Proton-Exchange Membrane Water Electrolysis
Source: Materials (Basel). 2024 Mar 14;17(6):1331. doi: 10.3390/ma17061331 (PMC10972082; doi:10.3390/ma17061331)
Supplement: Supplementary file 1 [file materials-17-01331-s001.zip › materials-2909430-supplementary.pdf]

Study of the Durability of Membrane Electrode Assemblies in Various Accelerated Stress Tests for Proton-Exchange Membrane Water Electrolysis

Zhengquan Su [1,2], Jun Liu [2,*], Pengfei Li [2] and Changhao Liang [2]

[1] Institute of Physical Science and Information Technology, Anhui University, Hefei 230601, China

[2] Key Laboratory of Materials Physics, Anhui Key Laboratory of Nanomaterials and Nanotechnology, Institute of Solid State Physics, Chinese Academy of Sciences, Hefei 230031, China

* Correspondence: jliu@issp.ac.cn; Tel.: +86-55165591320; Tel./Fax: +86-55165591434

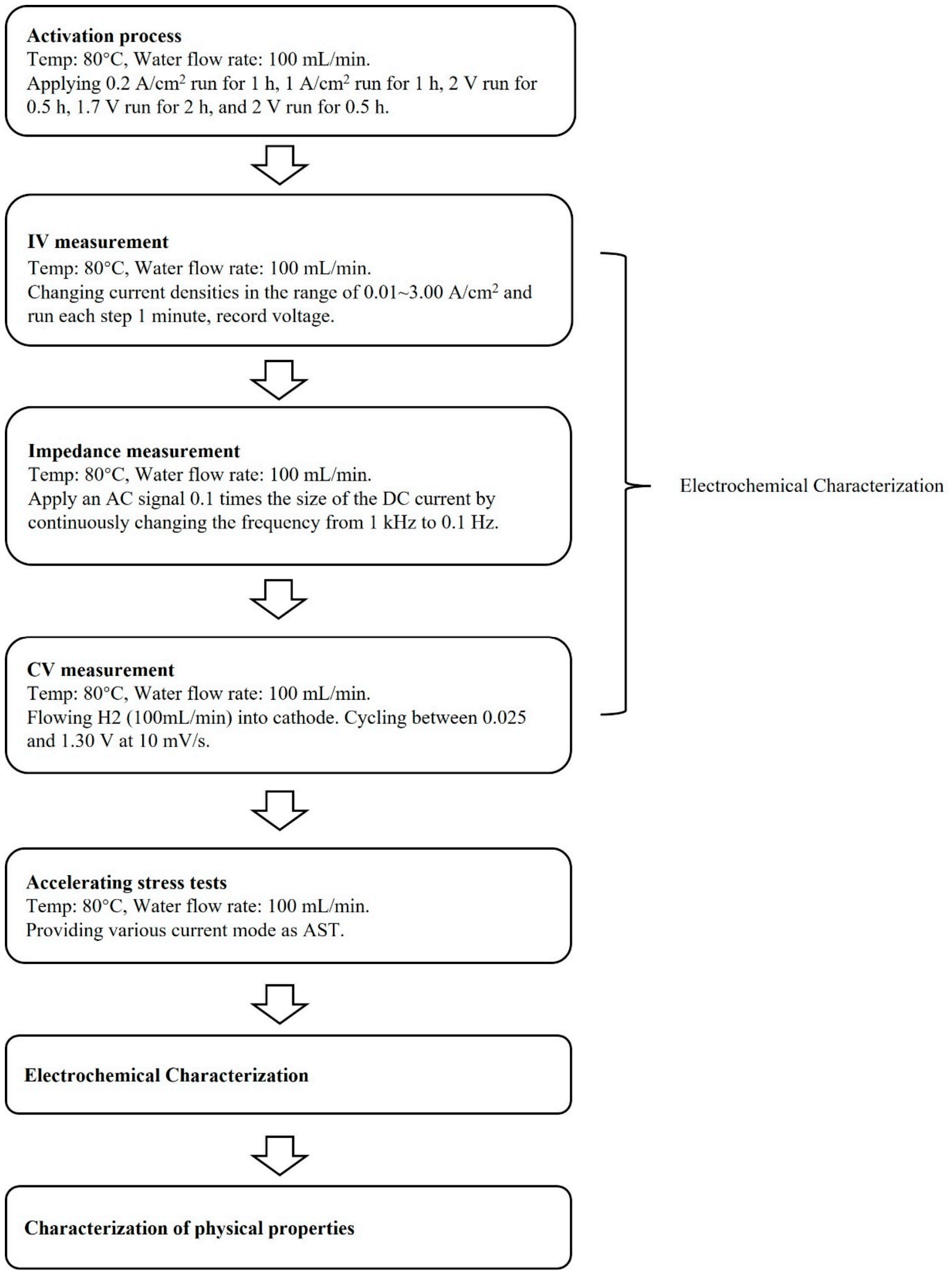

Figure S1. A graphical outline of accelerated stress tests.

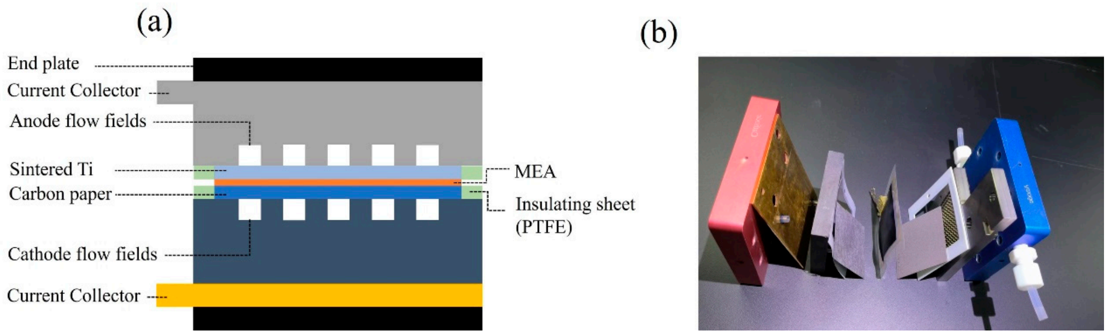

Figure S2. (a) Schematic diagram of the structure and (b) exploded view of the electrolyzer used in the investigation.

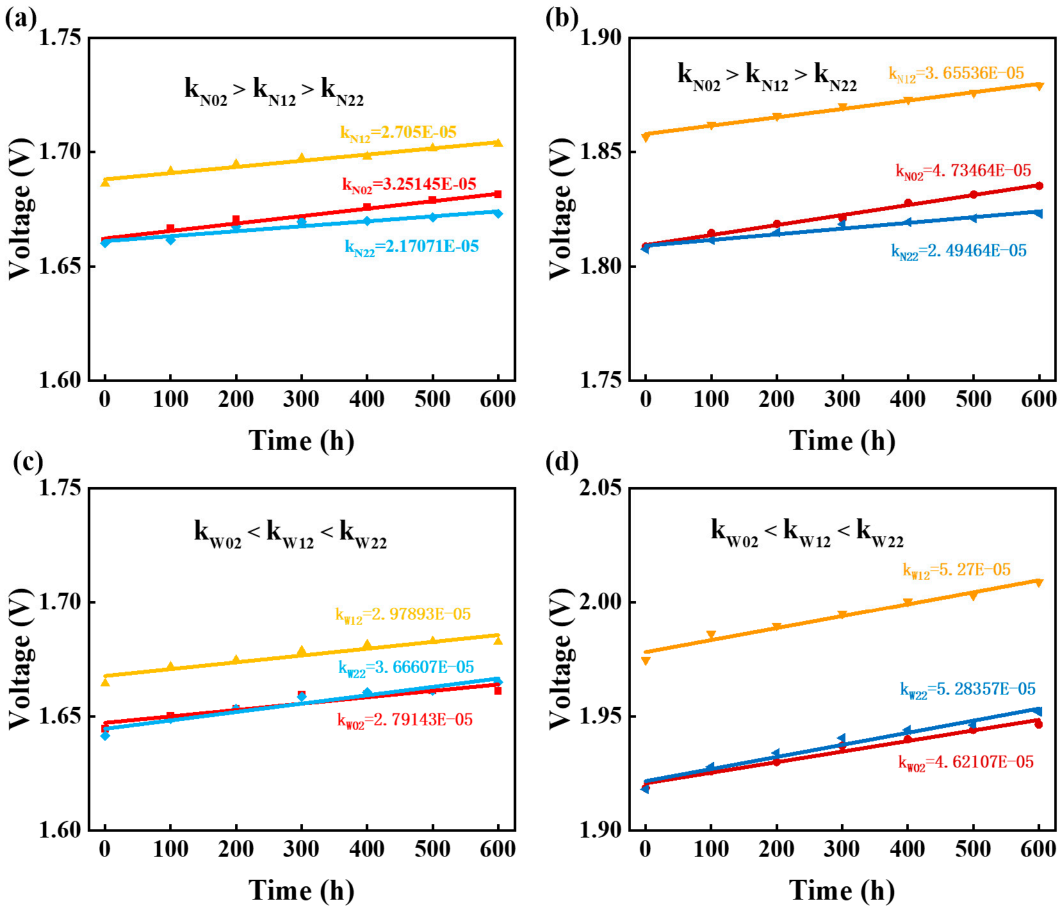

Figure S3. Degradation rate of narrow square wave mode in (a) 1 A/cm^2 and (b) 2 A/cm^2, and degradation rate in (c) 1 A/cm^2 and (d) 3 A/cm^2 for wide square wave mode, the slope k represents the degradation rate.

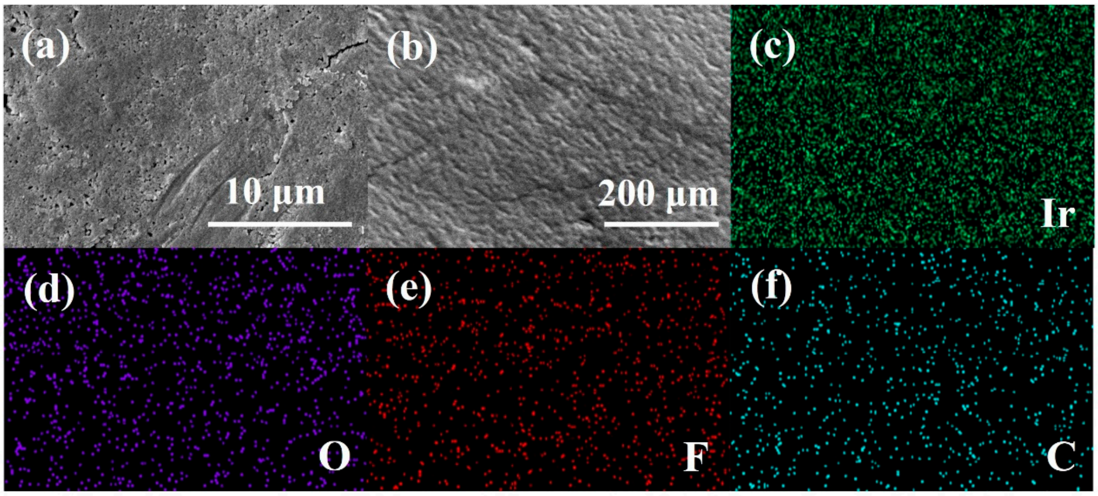

Figure S4. (a) High and (b) low magnification SEM images and (c–f) EDS mappings of the fresh MEA used in this investigation.

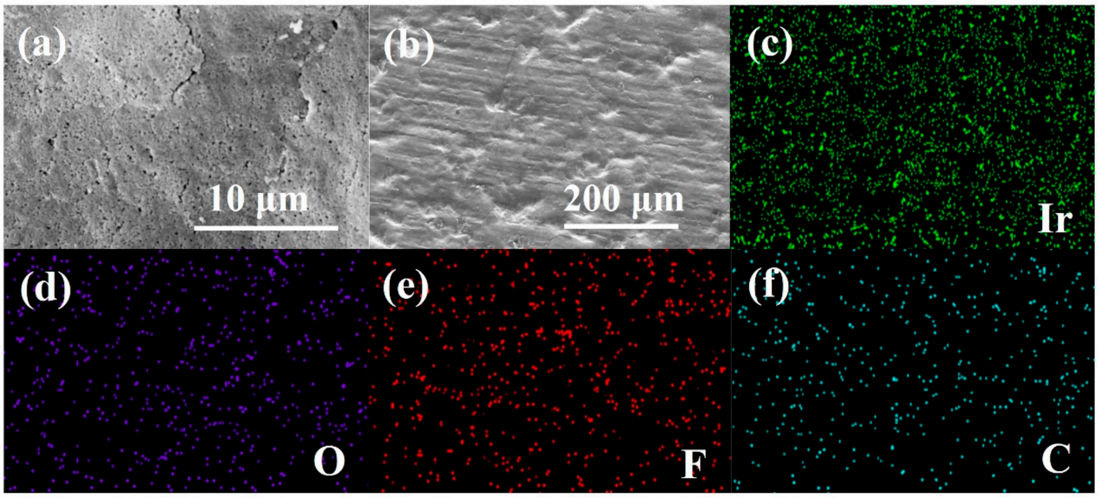

Figure S5. (a) High and (b) low magnification SEM images and (c–f) EDS mappings of the MEA after constant current mode AST C3.

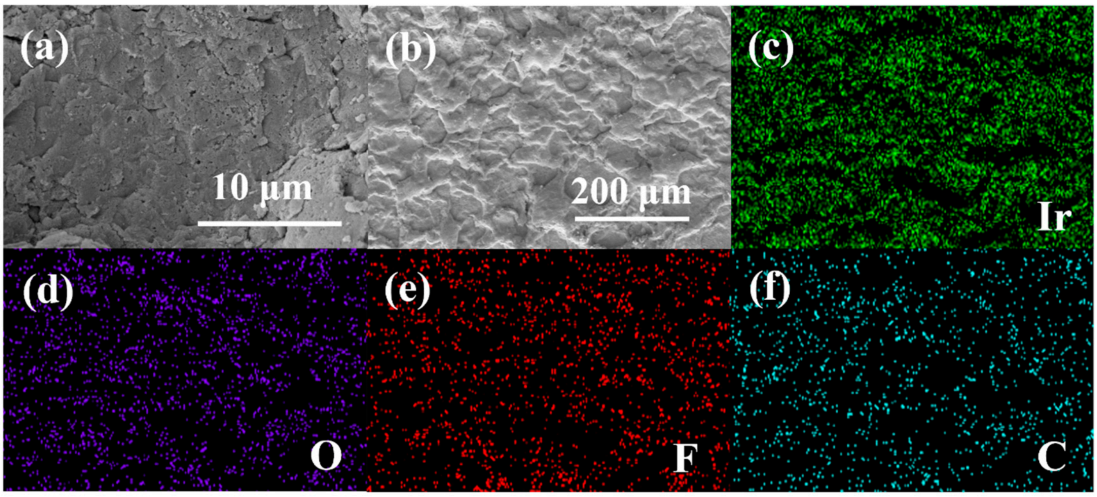

Figure S6. (a) High and (b) low magnification SEM images and (c–f) EDS mappings of the MEA after narrow square wave mode AST N02.

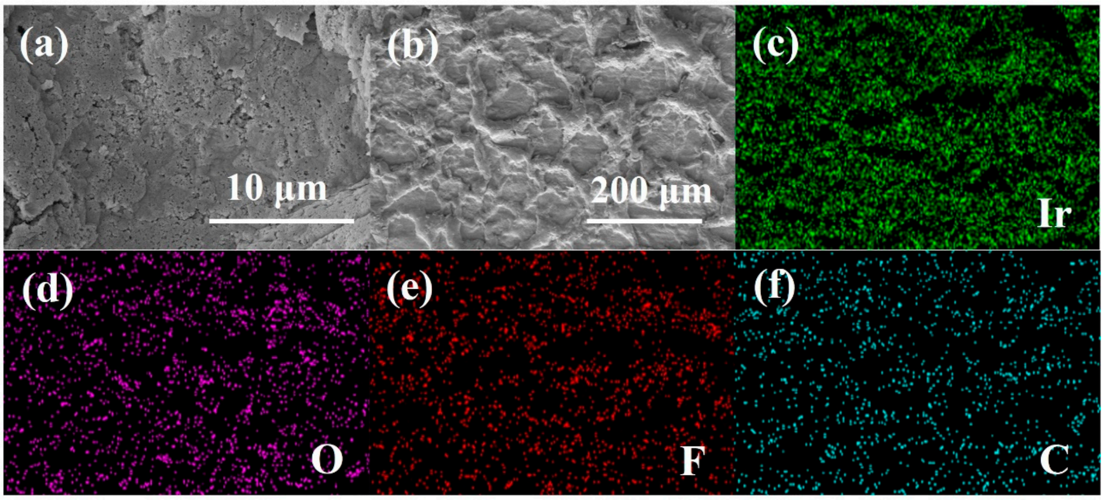

Figure S7. (a) High and (b) low magnification SEM images and (c–f) EDS mappings of the MEA after wide square wave mode AST W22.

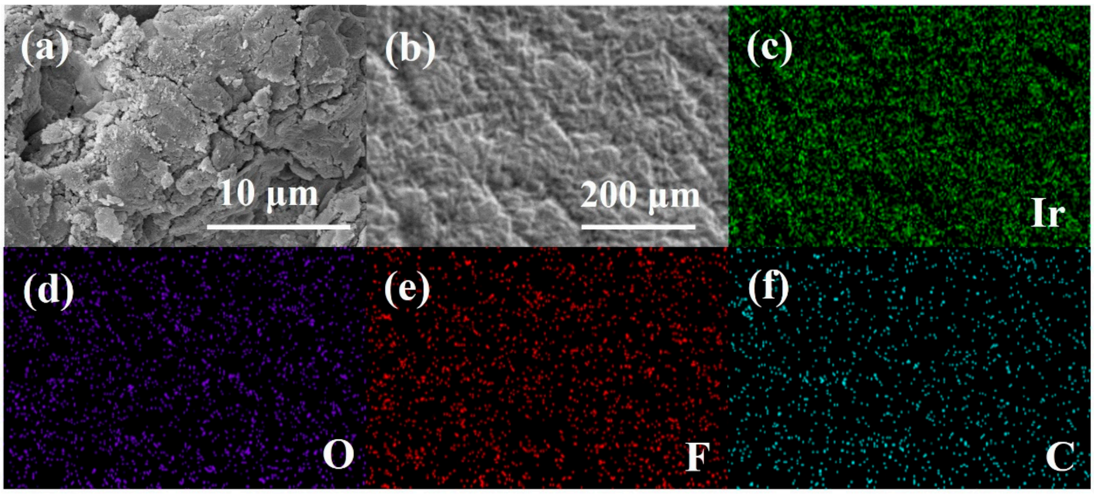

Figure S8. (a) High and (b) low magnification SEM images and (c–f) EDS mappings of the MEA after solar photovoltaic mode AST.

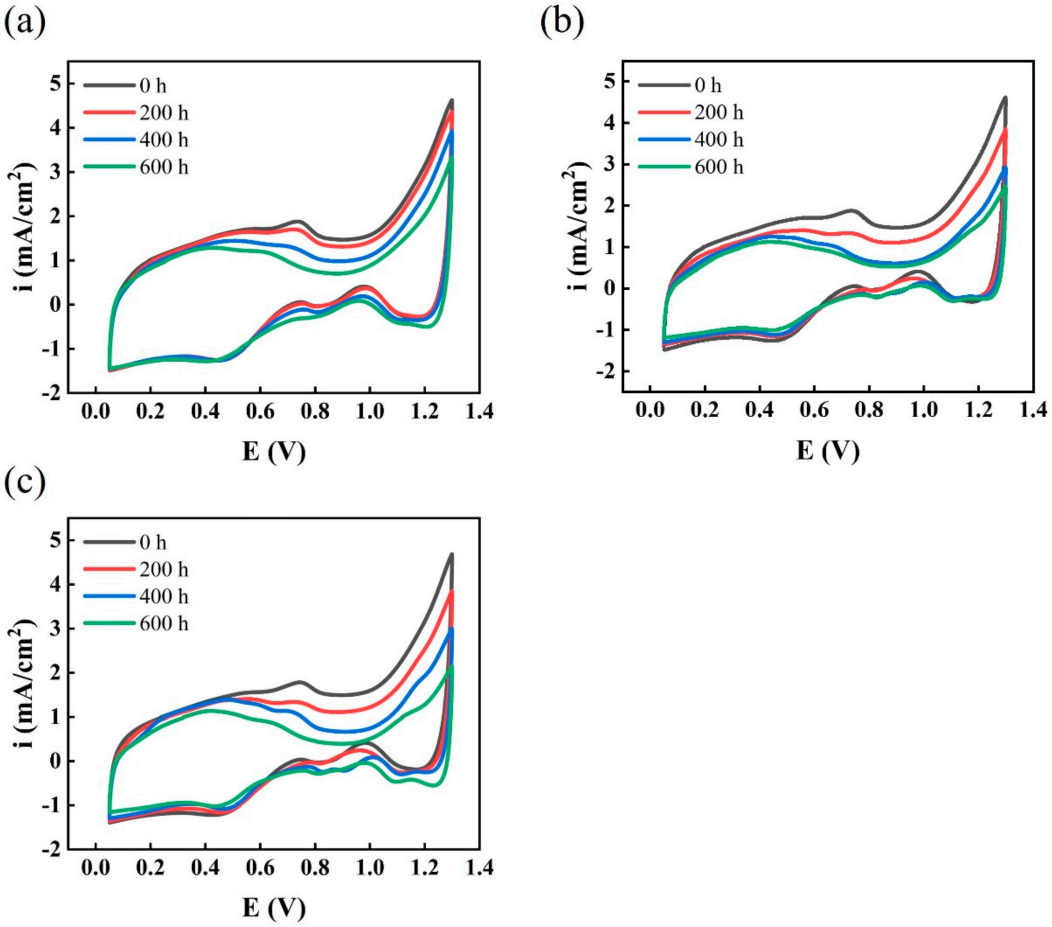

Figure S9. CV curves over time in (a) constant current density mode AST C3, (b) narrow square wave mode AST N02 and (c) wide square wave mode AST W22.

Table S1. Voltage at different times in constant current test.

	**C1**	**C2**	**C3**
0	1.66708	1.82256	2.01449
100	1.67477	1.81718	2.0088
200	1.67892	1.82071	2.01587
300	1.68	1.82287	2.01787
400	1.6803	1.82686	2.02618
500	1.68276	1.83179	2.03494
600	1.68246	1.83517	2.04032

Table S2. Voltage in 1 A/cm^2 and 2 A/cm^2 at different times in narrow square wave mode test.

	N02		N12		N22	
	1 A/cm^2	2 A/cm^2	2 A/cm^2	2 A/cm^2	1 A/cm^2	2 A/cm^2
0 h	1.66098	1.80869	1.68623	1.85658	1.66017	1.80755
100 h	1.66666	1.8146	1.69166	1.86198	1.66151	1.81154
200 h	1.67063	1.81863	1.69478	1.86571	1.66741	1.81484
300 h	1.66963	1.82093	1.69741	1.87011	1.66955	1.8185
400 h	1.67583	1.82786	1.69802	1.87285	1.66988	1.81942
500 h	1.67893	1.83142	1.70178	1.87591	1.67142	1.821
600 h	1.68142	1.83523	1.70365	1.87903	1.673	1.823

Table S3. Voltage in 1 A/cm^2 and 3 A/cm^2 at different times in wide square wave mode test.

	W02		W12		W22	
	1 A/cm^2	3 A/cm^2	1 A/cm^2	3 A/cm^2	1 A/cm^2	3 A/cm^2
0 h	1.64451	1.91857	1.66433	1.9745	1.64149	1.91807
100 h	1.65002	1.926	1.6717	1.98602	1.64874	1.92792
200 h	1.65314	1.92996	1.67462	1.98962	1.65319	1.93397
300 h	1.65925	1.93709	1.67872	1.99491	1.65856	1.94049
400 h	1.65955	1.9399	1.68141	2.00027	1.66052	1.94404
500 h	1.66119	1.94404	1.68277	2.00295	1.66121	1.94611
600 h	1.66098	1.94636	1.68249	2.00885	1.66495	1.9519

Table S4. Voltage at different times in Simulating solar fluctuation test.

	1 A/cm^2	2 A/cm^2	3 A/cm^2
0 h	1.61046	1.7563	1.89586
110 h	1.64144	1.7932	1.94432
220 h	1.65357	1.80284	1.95093
330 h	1.64858	1.79669	1.95048
440 h	1.65149	1.79789	1.94821
550 h	1.65556	1.80401	1.96056
660 h	1.66163	1.81044	1.98249
770 h	1.6591	1.81003	1.99477
880 h	1.66591	1.81835	1.99363
990 h	1.67099	1.8294	2.00641
1100 h	1.67564	1.83435	2.02057
1210 h	1.67359	1.83267	2.0244
1320 h	1.68765	1.85337	2.03804
1430 h	1.68599	1.85088	2.03452
